# Public knowledge and attitudes towards wastewater treatment works and plastic pollution in the Vhembe District Municipality, South Africa

**DOI:** 10.1371/journal.pone.0325236

**Published:** 2025-06-30

**Authors:** Khumbelo Mabadahanye, Mwazvita T.B. Dalu, Farai Dondofema, Linton F. Munyai, Tatenda Dalu

**Affiliations:** 1 School of Biology and Environmental Sciences, University of Mpumalanga, Nelspruit, South Africa; 2 Department of Geography and Environmental Sciences, University of Venda, Thohoyandou, South Africa; Indian Institute of Technology Delhi, INDIA

## Abstract

Concerns about plastic pollution and wastewater treatment plants (WWTPs) have grown in communities near these facilities due to their negative effects on the local environment and public health. This study surveyed 150 community members in the municipality of Thulamela local municipality (TLM) in the Vhembe district municipality (VDM), and the data was analysed using Microsoft Excel and SPSS. Female participants (57.3%) comprised a larger proportion than male participants (41.3%), and employed participants (32.0%) were more represented than retired ones (4.6%). Sixty–one percent (61%) of participants knew WWTPs, while 39% were not. Forty–eight percent expressed concerns about WWTPs due to odours, health risks, and property value loss due to the proximity to the plants. Seventy–seven percent of participants reported being aware of plastic pollution in wastewater, while 54% indicated receiving information about this issue. Spearman’s rank–order correlation analysis revealed significant relationships among variables (sociodemographic and environmental consciousness behaviour), with higher education levels negatively correlated with WWTP awareness, while awareness of WWTPs positively correlated with knowledge of plastic pollution. Participants showed interest in educational outreach and gaining more information about WWTPs and plastic pollution. Public education and awareness campaigns are essential to address community concerns and increase environmental awareness.

## Introduction

Wastewater treatment removes contaminants from sewage or wastewater and transforms them into effluent that can be reused for various purposes or safely returned to the water cycle with minimal environmental and human health impacts [1]. The primary purpose of wastewater treatment plants (WWTPs) is to eliminate potential pathogens and remove nutrients, such as nitrogen and phosphorus, along with readily biodegradable dissolved organic matter, suspended particles, and solid wastes, including plastics, from wastewater [2,3]. In addition to household wastewater, which includes human waste such as urine and faeces from disease–carrying individuals, WWTPs also process non–domestic contaminants like heavy metals, pesticides, and hydrocarbons that infiltrate the wastewater system through rainwater runoff from urban infrastructure, including buildings, highways, gardens, and parks [2,4,5].

Plastic pollution is a significant issue threatening human health and environmental sustainability in the twenty–first century [6,7]. When plastics are not properly managed, they pose serious risks to human health and the aquatic environment [8]. Plastics enter wastewater systems through various sources, such as urban runoff, improper waste disposal, and household wastewater containing microplastics from personal care products and synthetic textiles [8,9]. Microplastics are of particular concern due to their ability to interact with and bind to other contaminants commonly found in wastewater, such as heavy metals, pesticides, and hydrocarbons [10–12]. These interactions can facilitate the transport of harmful chemicals within the environment, increasing the risk of contamination [13,14].

Although WWTPs effectively remove many pollutants, some microplastics bypass treatment processes and are discharged into the environment through effluent [15]. Microplastics can also accumulate in sewage sludge, often processed and reused in agriculture or land application, further contributing to environmental contamination [9]. The fact that plastic litters persist in the environment for a long time and eventually decomposes into smaller debris, such as microplastics (MPs, < 5 mm) and nanoplastics (NPs, < 100 nm), through physical chemical and biological processes is especially concerning [16–19]. Because of their small size, global spread, and possible ecotoxicological impacts, MPs have drawn significant public attention [20]. Micro– and nanoplastics (MNPs) concentrations in wastewater treatment plants have been reported to range from as high as 680 μg L^−1^ in influents to as low as 24 μg L^−1^ in effluents [21]. While traditional WWTPs remove some MNPs, the removal is incomplete. Consequently, while WWTPs play a critical role in wastewater treatment, their current processes often fall short of effectively removing MNP pollutants [22]. As a result, the effluents discharged from these plants can still contain significant quantities of MNPs, emphasizing the need for specialized treatment or modifications in existing processes to improve MNP removal [23–25].

In addition to pollution challenges, the interplay of various domestic activities exacerbates environmental and infrastructure difficulties [26]. Improper disposal of wet wipes in toilets and fats, oils, and greases (FOGs) down kitchen sinks leads to large sewer obstructions known as “fatbergs” [27]. Similarly, sanitary products contribute to sewer blockages due to their swelling properties when saturated with fluid [28]. Routine domestic practices such as improper disposal of plastic wastes pose further challenges to wastewater systems and sanitation facilities, endangering public health and the environment. Environmental plastic pollution requires complex solutions due to its severe impacts, making it a challenging governance and restoration issue [29–31]. Developing countries, including South Africa, face additional burdens due to the significant costs associated with waste management operations [32,33]. The Vhembe district of the Limpopo province, which is one of South Africa’s poorest municipalities, demonstrates these challenges. The widespread presence of street vendors contributes to solid waste generation and littering in the area [34].

Globally, research has primarily focused on microplastics, while less attention has been given to macroplastics, despite their environmental significance [35]. Understanding community perceptions regarding plastic pollution and wastewater reuse is crucial for addressing knowledge gaps and promoting public acceptance of wastewater treatment technologies and resource recovery initiatives [36,37]. In some regions, a lack of awareness about plastic pollution and community safety related to wastewater reuse poses significant barriers, often contributing to the global failure of wastewater treatment systems, particularly when treated wastewater is used in agriculture [38,39]. Gender roles also influence perceptions, with traditional norms associating men with the breadwinner role and women with the caretaker role [40]. Behavioral patterns and decisions can be further explained by the Theory of Planned Behaviour (TPB), which emphasizes the role of intentions in determining behavior [41,42]. Additionally, fostering environmental literacy (EL) is essential for creating a society capable of responsibly addressing cultural, political, and social environmental challenges [43–45]. This study aimed to assess social perceptions, attitudes and knowledge gaps concerning WWTPs and plastic pollution within the local communities of TLM in Vhembe district, South Africa. This study hypothesises that residents of the Thulamela Municipality have limited awareness of WWTPs and plastic pollution, as well as their environmental impacts and potential effects on communities living near these plants. By investigating this topic, the study aims to enhance understanding of public knowledge and attitudes, which can inform targeted educational campaigns, policy development, and improved community engagement strategies to address these challenges effectively.

## 2. Methods

### 2.1 Research ethics

Ethical approval for this study was granted by the University of Mpumalanga Research Ethics Committee, number UMP/Dalu/1/2022. Before conducting the study, application documents were submitted to the VDM, seeking permission to do a survey regarding the public knowledge and attitudes towards wastewater treatment works WWTPs. The study was conducted only with the written consent of the participants as approved by the ethics committee. We ensured compliance with informed consent requirements and protected participant privacy by adhering to two common standards: (1) secrecy and (2) anonymity.

### 2.2 Study area

The study was conducted in the TLM (Table 1), located within the VDM. Nine villages within the Thulamela Local Municipality were selected for the study, where community members were interviewed (Table 2). The municipality, which is one of the five that form part of the Vhembe Biosphere Reserve (VBR) in Limpopo Province [46], has a population of 575,929 [47]. Geographically, it is located approximately between latitude 22° 57’ S and longitude 30° 29’ E [48].

**Table 1:** Demographics of selected local municipality within Vhembe District Municipality. Data Source: Statistics SA [47].

Population size	Sex ratio	Age Structure	Education	Number of Houses
575 929	Male (46.6%)	Young children (0–14 years) (31.8%)	No schooling (20 + years) (13.4%)	142 527
	Female (53.4%)	Working age population (15–64 years) (61.7%)	Higher education (20 + years) (13.9%)	
		Elderly (65 + years) (6.5%)		

**Table 2:** Villages selected for the survey and their coordinates.

Villages	Coordinates
Tswinga	23°01’29“S, 30°29’04”E
Thohoyandou Unit E (Hamagidi)	22°57’14“S, 30°29’12”E
Manini	22°59’20“S, 30°28’14”E
Tshilungoma	22°58’21“S, 30°30’07”E
Tshikweta	22°55’58“S, 30°30’43”E
Thivhulani	22°55’56“S, 30°28’02”E
Mbilwi	22°55’51“S, 30°28’07”E
Hamavhunda	22°56’31“S, 30°30’43”E
Makwarela	22°57’46“S, 30°29’46”E

### 2.3 Sampling and data collection

This study employed a qualitative methodology to collect data through in–person interviews with 150 community members older than 18 years across 10 villages within the Thulamela Local Municipality between June and July 2024. A structured questionnaire designed to incorporate closed– and open–ended questions assessed community awareness, attitudes, and perceptions regarding wastewater treatment plants (WWTPs) and plastic pollution (S1 Text). To ensure a representative sample and minimize selection bias, participant families were randomly selected, with only one individual from each family participating in the study. Each interview lasted approximately 10–15 minutes, and the questionnaire was organized into distinct thematic sections: sociodemographic information (questions 1–4), awareness and knowledge of WWTPs (questions 5–11), perceptions of WWTPs (questions 12–23), willingness to engage with WWTP–related initiatives (questions 24–29), and perceptions of WWTP effectiveness (questions 30–45). The questionnaire was designed to be concise and straightforward, ensuring clarity and preventing respondent fatigue. The study’s purpose and methodology were explained to participants beforehand, and instructions for completing the questionnaire were provided. While the survey was primarily in English, clarifications were offered in TshiVenda, the local language, to enhance comprehension. Respondents were assured of anonymity, and the researcher remained available to address any questions or misunderstandings during the interviews. Data collection continued until saturation was achieved after completing 150 questionnaires, at which point no new or significant insights were identified [49]. The structured and systematic approach to sampling and data collection ensured the reliability and validity of the study findings.

### 2.4 Data analysis

The data obtained from the survey were analysed using Microsoft Excel and IBM SPSS Statistics software. Microsoft Excel was used to organise the data and generate charts and graphs, providing clear visual representations of public knowledge and attitudes regarding wastewater treatment plants (WWTPs) and plastic pollution. IBM SPSS Statistics was employed for statistical analysis. Spearman’s rank–order correlation was used to examine the relationships between sociodemographic variables (e.g., gender, age, education), awareness of WWTPs, and environmentally conscious behaviours, such as attitudes toward plastic pollution. This method was chosen because it is non–parametric and does not require data to meet assumptions of normality or linearity.

## 3. Results

### 3.1 Sociodemographic

One hundred and fifty communities of households of TLM completed hard copies of questionnaires. Based on the collected data, most respondents were female, accounting for 57.3% (*n* = 86), compared to 41.3% (*n* = 62) male respondents; 1.3% (*n* = 2) of participants chose not to disclose their gender. The age group of 25–34 years was the most represented, making up 31.3% of respondents, while the 55 + age group was the least represented at 9.3%, with 1.3% of respondents preferring not to specify their age. Regarding educational background, 23.3% of respondents held a degree, 19.3% had a postgraduate degree, 16.6% possessed a diploma, 1.3% had completed only primary school, and 7.3% were uneducated. Employment status data revealed that 32.0% of respondents were employed, followed by 22.6% who were unemployed, 22.0% who were self–employed, 18.6% who were still studying, and 4.6% who were retired (Table 3).

**Table 3:** Sociodemographic of local communities of the Thulamela Local Municipality.

Variables	Number	Percentage (%)
***Gender***		
Male	62	41.3
Female	86	57.3
Prefer not to say	2	1.3
***Age group***		
18–24	44	29.3
25–34	47	31.3
35–44	26	17.3
45–54	17	11.3
55+	14	9.3
Prefer not to say	2	1.3
***Education level***		
Uneducated	11	7.3
Primary school	2	1.3
High School	20	13.3
Certification	28	18.6
Diploma	25	16.6
Degree	35	23.3
Postgraduate degree	29	19.3
***Employment Status***		
Student/Unemployed	28	18.6
Self–employed	33	22.0
Retired	7	4.6
Unemployed	34	22.6
Employed	48	32.0

### 3.2 Awareness and knowledge of wastewater treatment plant and their role in plastic pollution

**Fig 1:**
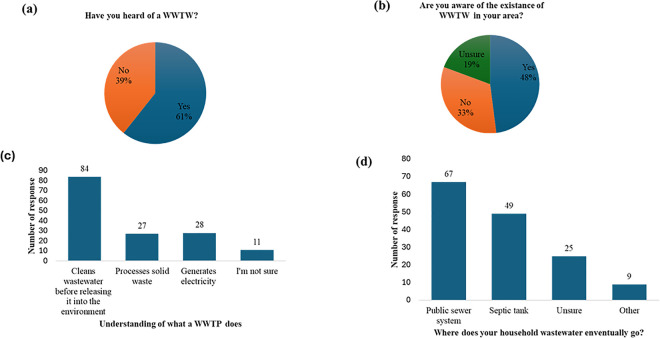
Responses regarding awareness and knowledge of wastewater treatment plants (WWTPs): Familiarity, awareness and distance to WWTPs.

The results revealed that 61% of respondents had knowledge of WWTPs, while 39% had not (Fig 1a). However, only 48% of participants reported being aware of WWTPs in their area, with 33% unaware of nearby facilities and 19% unsure (Fig 1b). When participants were asked about the role of WWTPs, 84 respondents correctly identified that WWTPs clean wastewater before releasing it into the environment. However, some confusion was also noted, as 27 participants believed WWTPs process solid waste, 28 stated they generate electricity, and 11 were unsure (Fig 1c).

To assess perceptions of plastics in wastewater, participants were asked about the fate of their household wastewater, which can be a pathway for plastic contamination in WWTPs. Sixty–seven participants stated their household wastewater enters a public sewer system, 49 reported using septic tanks, and 25 were unsure (Fig 1d).

### 3.3 Perceptions of wastewater treatment plants

**Fig 2:**
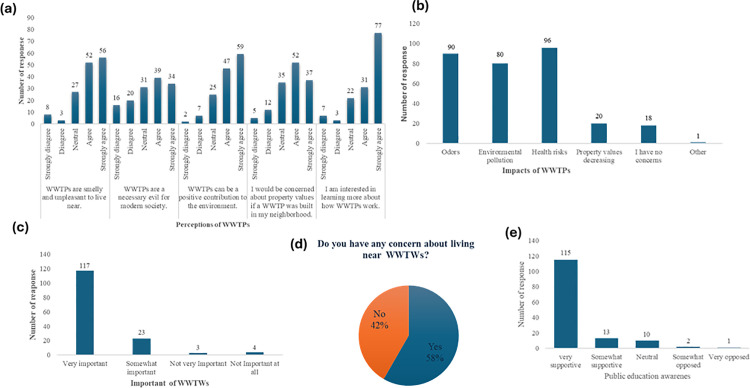
Participants’ perceptions of wastewater treatment plants (WWTPs): Impacts, importance, concerns about distance, and support for public education awareness.

Forty–six participants strongly agreed that WWTPs are unpleasant and produce smells, making it challenging to live nearby, while only three participants strongly disagreed with this statement. Additionally, 39 participants agreed with the perception that WWTPs are a “necessary evil” for modern society, though 16 respondents strongly disagreed, highlighting a division in perceptions about their importance. When asked if WWTPs can positively impact the environment, 59 respondents strongly agreed, emphasizing awareness of the environmental benefits these WWTPs can provide, while only two respondents strongly disagreed. Furthermore, 52 participants expressed concern about the potential impact on property values if a WWTP were constructed in their neighbourhood, indicating a worry about the infrastructure. Interest in understanding how WWTPs operate was high among participants, with 77 respondents expressing strong interest in learning more, compared to just 3 respondents who were uninterested (Fig 2a).

Most participants (*n* = 117) recognised the importance of WWTPs to their community, though four respondents believed these treatment works were not important at all (Fig 2c). Fifty–eight participants expressed concerns about living near WWTPs, while 42 indicated no concerns (Fig 2d). Regarding concerns about living near WWTPs, 96 participants expressed worry about potential health risks, and 90 were troubled by the odours emitted. Additionally, 80 respondents highlighted the contribution of WWTPs to environmental pollution, while only 18 participants indicated they had no concerns about living close to these facilities (Fig 2b). Most participants (*n* = 115) expressed strong support for increased public education awareness about the safety and benefits of WWTPs, with only one participant opposing such awareness initiatives (Fig 2e). This indicates that community members value understanding wastewater treatment plants’ safety measures and benefits.

### 3.4 Willingness to engage

**Fig 3:**
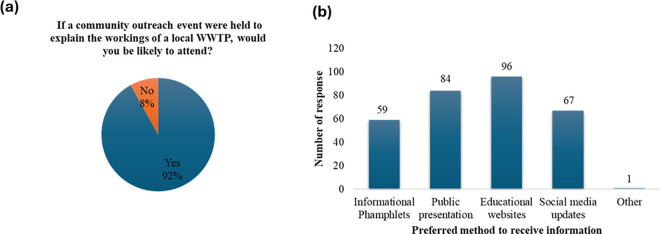
Community interest in outreach initiatives and preferred methods for receiving information about wastewater treatment plants (WWTPs).

Ninety–two percent of participants indicated that they would likely attend community outreach events aimed at explaining the workings of local WWTPs, while 8% said they would not be interested (Fig 3a). Regarding preferred methods for receiving information about WWTPs, 96 participants chose educational websites, 84 preferred public presentations, 67 selected social media updates, and 59 chose informational pamphlets (Fig 3b). The responses suggest high community interest in understanding how these WWTPs operate.

### 3.5 Perceptions about the effectiveness of wastewater treatment plants in addressing plastic pollution

**Fig 4:**
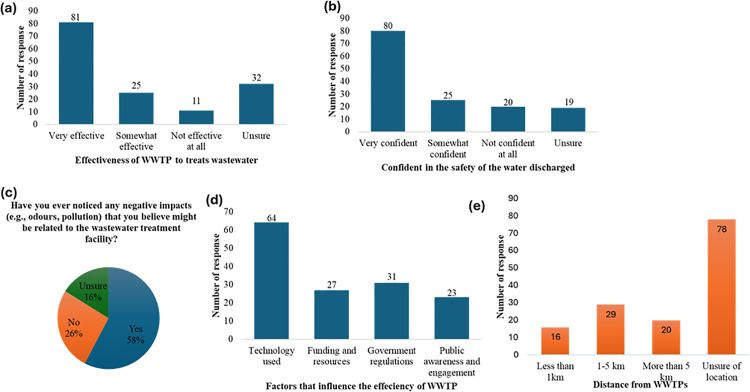
Perceptions of wastewater treatment plants (WWTPs) effectiveness, confidence in discharged water safety, observations of negative impacts, and factors influencing treatment efficiency.

In assessing the factors influencing the efficiency of WWTPs, 64 participants highlighted the significance of the technology employed, while 31 emphasized the role of government policies and regulatory frameworks. Additionally, 27 participants identified funding and resource availability as key determinants, whereas 23 understood the importance of public awareness and community engagement. (Fig 4d). This response highlights the complex relationship between WWTP efficiency and community involvement, proper governance, and technological developments.

When participants were asked about the effectiveness of WWTPs in treating wastewater, 81 participants rated them as “very effective,” while 32 participants expressed uncertainty about their effectiveness (Fig 4a). However, confidence in the safety of water discharged from WWTPs was relatively high, with 80 participants indicating confidence in its safety, while 19 participants were unsure (Fig 4b). Despite this, 58% of respondents reported noticing negative impacts they associated with WWTPs, such as odours or pollution, while 26% stated they had not observed any impacts, and 16% were unsure (Fig 4c).

Factors influencing WWTP efficiency were also assessed to explore the relationship between community perceptions and the issue of plastic pollution. Among the respondents, 64 participants emphasized the importance of the technology used in treatment processes, 31 highlighted the role of government and regulations, 27 pointed to funding and resources, and 23 emphasized the need for public awareness and engagement (Fig 4d). These factors collectively suggest that technological advancements and adequate resources are critical for addressing challenges such as removing microplastics and other contaminants in WWTPs.

The proximity of households to WWTPs was investigated to understand potential exposure to environmental and health impacts caused by these facilities. While 16 respondents reported living less than 1 km from a WWTP, 29 indicated they lived between 1–5 km away, 20 stated they resided more than 5 km away, and 78 were unsure of their distance (Fig 4e). A spatial analysis of the proximity of households to WWTPs (Fig 4e), mapping the relationship between household location and WWTPs to assess the extent of perceived or real impacts on nearby communities.

### 3.6 Perceptions of plastic pollution

**Fig 5:**
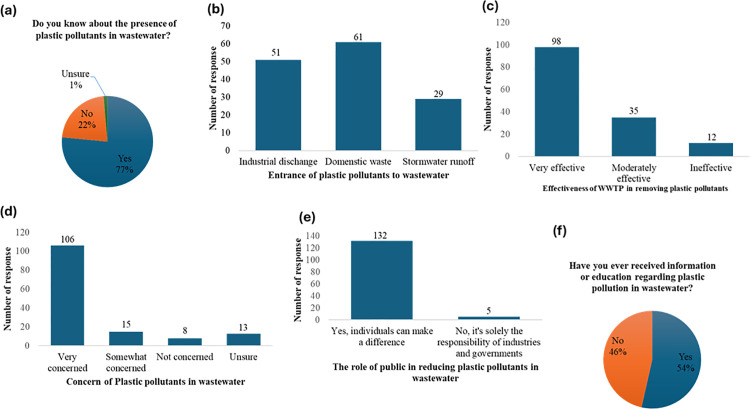
Public awareness, sources, concerns, and perceived effectiveness of wastewater treatment plants (WWTPs) in addressing plastic pollution.

Most participants (77%) indicated awareness of plastic pollutants in wastewater, while 22% said they were unaware, and 1% were unsure (Fig 5a). When asked how plastic pollutants enter wastewater systems, 61 participants identified domestic waste, 51 pointed to industrial discharge, and 29 mentioned stormwater runoffs as a key source (Fig 5b). Ninety–eight participants regarded WWTPs as very effective at removing plastic pollutants. Meanwhile, 35 participants considered them moderately effective, and 12 perceived them as ineffective (Fig 5c). Concern about plastic pollution was high, with 106 participants expressing worry, while 13 were unsure (Fig 5d). Regarding the public’s role in reducing plastic pollution, 132 participants agreed that individuals can make a difference, whereas 5 believed it is solely the responsibility of industries and governments (Fig 5e). Lastly, when asked if they had ever received information or education about plastic pollution in wastewater, 54% of participants said yes, while 46% said no (Fig 5f).

### 3.7 Relationship between education, awareness and environmental consciousness behaviour

Education showed a significant negative correlation with awareness and knowledge about WWTPs (*r* = − 0.31, *p *< 0.01), suggesting that individuals with higher education levels may have lower awareness. A significant positive correlation was observed between awareness and knowledge of WWTPs and plastic pollution information *(r* = 0.45, *p* < 0.01), indicating that greater awareness is associated with increased knowledge of plastic pollution issues. Awareness of WWTPs was positively correlated with concern near WWTPs (*r *= 0.122, *p* < 0.05) and with knowledge of plastic pollutants (*r* = 0.12, *p* < 0.05). Learning about WWTP functions strongly correlated with willingness to engage in WWTP–related activities (*r* = 0.62, *p* < 0.01). Plastic pollution information was positively associated with willingness to engage (*r* = 0.35, *p *< 0.01) and knowledge of plastic pollutants (r = 0.29, *p* < 0.01). These results showed the relationship that exist between awareness, education, and environmental consciousness behaviour, especially regarding wastewater treatment and plastic pollution (Table 4).

**Table 4:** Relationship between sociodemographic variables and environmental consciousness behaviour towards plastic pollution and wastewater treatment plants (WWTP).

	Sociodemographic			Environmental consciousness behaviour							
	Age	Gender	Education	Heard of WWTP	Aware of WWTP	Concern near WWTP	Willingness of engage	Learning WWTP function	WWTP Negative Impacts	Knowledge of plastic pollutants	Plastic pollution information
Heard of WWTP	0.09	–0.06	–0.31^**^	1.00	0.37^**^	0.25^**^	0.10	0.12	0.34^**^	0.15	0.45^**^
Aware of WWTP	0.05	–0.03	–0.20^*^	0.37^**^	1.00	0.12	0.01	0.11	0.22^**^	0.12	0.39^**^
Concern near WWTP	–0.06	0.06	–0.11	0.25^**^	0.12	1.00	0.07	0.10	0.10^*^	0.14	0.03
Willingness of engage	–0.05	–0.08	–0.19^*^	0.10	0.05	0.07	1.00	0.62^**^	0.10	0.09	0.19^*^
Learning WWTP function	–0.07	–0.05	–0.20^*^	0.12	0.11	0.10	0.62^**^	1.00	0.17^*^	0.09	0.08
WWTP Negative Impacts	–0.08	–0.04	–0.26^**^	0.34^**^	0.22^**^	0.19^*^	0.10	0.17^*^	1.00	0.15	0.35^**^
Knowledge of plastic pollutants	0.11	0.06	–0.04	0.15	0.12	0.14	0.09	0.01	0.15	1.00	0.29^**^
Plastic pollution information	0.04	–0.08	–0.13	0.45^**^	0.39^**^	0.03	0.19^*^	0.08	0.35^**^	0.29^**^	1.00

** Correlation is significant at the 0.01 level (*p* < 0.01). ^*^ Correlation is significant at the 0.05 level (*p *< 0.05).

## 4. Discussion

The study assessed public knowledge and attitudes towards WWTPs and the removal of plastic pollutants in the Vhembe District, South Africa. The hypothesis that public knowledge about WWTPs and plastic pollution would be limited was rejected, as most respondents demonstrated awareness of both topics and their impacts on the environment and communities near WWTPs. Survey findings revealed higher female participation than males, consistent with Mashamba *et al*. [50], where females comprised most participants. This trend aligns with Gender Role Theory, which attributes participation to traditional roles as caregivers and water gatherers, driving women’s engagement with environmental issues [40,51].

Age distribution analysis indicated that younger participants, mainly 25–34 years, were more engaged than older age groups. This trend supports the Environmental Literacy (EL) concept, which suggests that younger individuals may exhibit more significant concern for environmental issues due to increased exposure to environmental education during formative years [52]. The increased participation of younger respondents emphasizes the importance of focusing on youth in environmental campaigns to ensure sustained support for future initiatives.

Participants’ educational backgrounds varied, with a notable proportion possessing higher education qualifications, including degrees and postgraduate qualifications. However, those with lower levels of education may rely more on community outreach for their environmental knowledge. Previous research by Dalu *et al*. [53] suggests that higher education levels significantly influence environmental concerns and behaviour. Individuals with advanced education demonstrate greater pro–environmental behaviour due to their enhanced understanding of environmental issues. Similarly, Zhu *et al*. [54] found that individuals with bachelor’s degrees had higher knowledge levels about water–related issues than those in other educational groups.

The study revealed a significant variation in participants’ awareness and understanding of WWTPs and their role in managing plastic pollution. While some participants demonstrated familiarity with WWTPs’ functions, others were less informed, highlighting a gap in public awareness regarding the treatment processes and their environmental implications. This variation in understanding is crucial, especially when considering the impact of plastic pollution in wastewater systems. Participants expressed concerns not only about health risks but also about the presence of plastic pollution in WWTPs. These concerns resonate with findings from Hachi *et al*. [55], who identified odorous emissions as a key issue near WWTPs. The negative perceptions surrounding plastic pollution in WWTPs are significant, as they underscore the growing need to address community concerns about treating plastic waste in these facilities.

Perceptions of WWTP’s effectiveness in removing plastic pollutants were mixed. Some participants expressed confidence in the treatment process, believing that plastics were sufficiently filtered out. In contrast, others voiced concerns about the persistence of plastic particles in the effluent and their potential to pollute downstream environments. This is consistent with the findings of Mason *et al.* [56], which demonstrated that microplastics persist in treated effluent, posing significant risks to both human health and the environment. Moreover, a recent study by Mabadahanye *et al.* [22] further emphasised that WWTPs globally continue to release effluent–containing microplastics, highlighting the ongoing challenges of mitigating plastic pollution in wastewater systems. These findings reveal that, despite some trust in the treatment process, there is considerable uncertainty regarding its ability to fully eliminate plastic pollution. This underscores the urgent need for enhanced treatment technologies and clearer communication to address public concerns and mitigate the risks associated with plastic pollutants.

While participants demonstrated widespread awareness of plastic pollution in wastewater, many expressed concerns about its environmental impact. However, a significant gap in public education was identified, with many respondents reporting insufficient information regarding plastic pollution and its broader effects. This underscores the need for targeted educational campaigns to raise awareness and encourage environmentally responsible behaviours. However, formal education alone may not fully address the issue. Within the community, alternative methods for observing the impacts of plastic pollution exist, such as reduced crop yields in areas where effluent is used for irrigation, especially in regions where plastic accumulation is more prominent. The spatial distribution of plastic pollution may also be evident in certain activities or crops that are disproportionately affected, leading to visible signs of plastic contamination in agricultural products. A study by Zhou *et al*. [57] revealed that polypropylene microplastics (PP–MPs) significantly negatively impact crop growth and quality, further emphasizing the need for broad approaches to reduce plastic pollution at both the community and systemic levels. The Theory of Planned Behavior (TPB) supports this perspective, by emphasizing that knowledge and attitudes plays a crucial role in shaping individual actions and decision–making [58].

The study also demonstrated participants’ willingness to engage in community outreach activities, highlighting the importance of public involvement in environmental management efforts. According to Naughton and Hynds [59], understanding public perspectives is essential for sustainable environmental practices. Participants preferred diverse communication platforms, such as instructional websites, social media posts, and informational booklets, emphasizing the need for tailored communication strategies to reach various demographic groups effectively.

## 5. Conclusions

This study indicates that local communities have a moderate level of awareness and engagement regarding WWTPs’ role in environmental management. even though there are still some significant knowledge gaps. While WWTPs are widely recognized for their positive environmental impacts, participants expressed varying concerns. Some were particularly worried about potential health risks associated with wastewater treatment, while others highlighted unpleasant odours as a significant issue. Additionally, a few participants expressed concerns about potential property loss due to nearby WWTP operations. The study also emphasizes how age, gender, and educational background influence public knowledge and engagement with issues regarding WWTP. Higher education levels were found to have a substantial impact, as participants demonstrated a greater understanding of WWTP operations.

The findings indicate that the public is highly interested in accessing clear and understandable information regarding WWTP operations, especially their benefits, safety protocols, and efforts to reduce plastic pollution. Participants strongly supported educational outreach, preferring various communication channels, including websites, social media, and public presentations, to foster greater understanding and acceptance. However, as the Theory of Planned Behavior (TPB) highlights, knowledge and attitudes strongly influence public actions. This suggests that while formal education plays a significant role, the broader cultural context and individual beliefs also impact the effectiveness of educational initiatives. The theory of gender roles may provide insight into how societal expectations and roles influence community perceptions of WWTP operations and environmental issues. Environmental literacy is critical in shaping the public’s understanding and engagement with sustainable water management practices. To bridge the knowledge gap and promote community engagement, future projects should prioritize open communication while integrating culturally relevant approaches and emphasizing the importance of environmental literacy and personal responsibility. These strategies could contribute to overcoming community concerns, enhancing environmental awareness, and fostering positive perceptions of WWTPs. Local municipalities and stakeholders should implement educational awareness campaigns to increase community understanding of the adverse effects of plastic pollution and the importance of wastewater treatment. Residential developments should be prohibited by municipalities close to WWTPs because of the odours emitted by these plants, which can harm the health of those living nearby.

## Supporting information

S1 Text Questionnaire used in the study within the Vhembe District, South Africa. (DOCX)
