# Supplementary material for: Public knowledge and attitudes towards wastewater treatment works and plastic pollution in the Vhembe District Municipality, South Africa
Source: PLoS One. 2025 Jun 30;20(6):e0325236. doi: 10.1371/journal.pone.0325236 (PMC12208450; doi:10.1371/journal.pone.0325236)
Supplement: S1 Text — (DOCX) [file pone.0325236.s001.docx]

**Text S1.** Study questionnaire

This questionnaire aims to understand public knowledge and attitudes towards wastewater treatment works (WWTPs). Your honest responses will be crucial in shaping future communication and engagement efforts.

As part of the University of Mpumalanga, PhD programme, I, Khumbelo Mabadahanye, am conducting research entitled “Public knowledge and attitudes towards wastewater treatment works (WWTPs) and removing plastic pollutants in the Vhembe District, South Africa”. If you over 18 years old and live within the Vhembe District, you are invited to complete the following survey which should take you approximately 10-15 minutes.

The project is supervised by Dr Mwazvita Dalu and Dr Tatenda Dalu. Should you have any questions or concerns please feel free to contact either of us. Dr Mwazvita Dalu: [mwazvita.dalu@ump.ac.za](mailto:mwazvita.dalu@ump.ac.za), Dr Tatenda Dalu: [tatenda.dalu@ump.ac.za](mailto:tatenda.dalu@ump.ac.za) or Khumbelo Mabadahanye: mabadahanyek[@gmail.com](mailto:tshifura.rudzani@gmail.com).

Please note the following before completing the questionnaire.

- This questionnaire is completely voluntary,
- You must be 18 years or older to complete the survey,
- All answers will remain anonymous, with no way to identify the respondent,
- You are free to exit the questionnaire without having your answers recorded, simply by exiting the questionnaire at any time,
- The information collected will be used for publication and by answering the questionnaire, you give consent for your results to be used for this research.
- You are entitled to view the results of this study, upon completion (December 2024). You may do so by contacting myself, Dr M Dalu or Dr T Dalu.

Please answer the following questions, selecting the answer which most applies to you.

**Part 1: Demographics**

1. Age:

- 18-24
- 25-34
- 35-44
- 45-54
- 55+
- Prefer not to answer

1. Gender:
   - Male
   - Female
   - Non-binary
   - Prefer not to say
2. Highest level of education completed:
   - Uneducated
   - Primary school
   - High School
   - Certificate
   - Diploma
   - Degree
   - Postgraduate degree

4. Employment status

- - - Student/Unemployed
    - Self-employed
    - Retired
    - Unemployed
    - Employed

**Part 2: Awareness and Knowledge**

1. Have you ever heard of a wastewater treatment work (WWTP)?
   - Yes
   - No
2. If yes, what do you understand a WWTP to be? (Please answer in your own words)

…………………………………………………………………………………………………………………………………………………………………………………………………………………………………………………………………………………………………………………………………………………………………………

1. If you answered yes to question 1, what is your understanding of what a wastewater treatment works does? (**Select all that apply**)
   - Cleans wastewater before releasing it back into the environment
   - Processes solid waste
   - Generates electricity
   - I'm not sure
2. In your opinion, how important is it for a community to have a functioning WWTP?
   - Very important
   - Somewhat important
   - Neutral
   - Somewhat unimportant
   - Not important at all
3. Where does your household wastewater eventually go? (**Select all that apply**)
   - Public sewer system
   - Septic tank
   - Unsure
   - Other (please specify): ……………………………………………………….
4. Are you aware of the existence of wastewater treatment works in your community?
   - Yes
   - No
   - Unsure
5. How close do you live to a wastewater treatment work (if aware)?
   - Less than 1 km
   - 1-5 km
   - More than 5 km
   - Unsure of location

**Part 3: Perceptions**

1. Please rate your level of agreement with the following statements: (Use a scale of 1 - Strongly Disagree, 2 - Disagree, 3 - Neutral, 4 - Agree, 5 - Strongly Agree)

| **Variable** | **1 - Strongly Disagree** | **2 - Disagree** | **3 - Neutral** | **4 - Agree** | **5 - Strongly Agree** |
| --- | --- | --- | --- | --- | --- |
| WWTPs are smelly and unpleasant to live near. |  |  |  |  |  |
| WWTPs are a necessary evil for modern society. |  |  |  |  |  |
| WWTPs can be a positive contribution to the environment. |  |  |  |  |  |
| I would be concerned about property values if a WWTP was built in my neighborhood. |  |  |  |  |  |
| I am interested in learning more about how WWTPs work. |  |  |  |  |  |

1. Do you have any concerns about the potential impact of WWTPs on the environment or public health?

…………………………………………………………………………………………………………………………………………………………………………………………………………………………………………………………………………………………………………………………………………………………………………

1. How important do you believe wastewater treatment works are for our community?
   - Very important
   - Somewhat important
   - Not very important
   - Not important at all
2. Do you have any concerns about living near a wastewater treatment works? (**Select all that apply**)
   - Odors
   - Environmental pollution
   - Health risks
   - Property values decreasing
   - I have no concerns
   - Other (please specify):………………………………………………………..
3. If you answered yes to question 3, how would you feel about increased public education efforts regarding the safety and benefits of wastewater treatment works?
   - Very supportive
   - Somewhat supportive
   - Neutral
   - Somewhat opposed
   - Very opposed
4. Which of the following words or phrases best describe what comes to mind when you think of wastewater treatment works? (**Choose all that apply**)
   - Necessary
   - Unpleasant odor
   - Environmental protection
   - Public health risk
   - Outdated technology
   - Other (Please specify): ……………………………………………………….
5. Do you have any concerns about living near a wastewater treatment work?
   - Yes
   - No
6. If you answered yes to question 7, please elaborate on your concerns:

…………………………………………………………………………………………………………………………………………………………………………………………………………………………………………………………………………………………………………………………………………………………………………

**Part 4: Willingness to Engage**

1. If a community outreach event were held to explain the workings of a local WWTP, would you be likely to attend?
   - Yes
   - No
2. Would you be interested in learning more about how wastewater treatment works function?
   - Yes
   - No
3. What is the preferred method for you to receive information about wastewater treatment works? (**Choose all that apply**)
   - Informational pamphlets
   - Public presentations
   - Educational websites
   - Social media updates
   - Other (Please specify): …………………………………………………………
4. How interested would you be in learning more about how wastewater treatment works function?
   - Very interested
   - Somewhat interested
   - Not interested
5. Do you have any suggestions on how wastewater treatment works can better engage with the community?

……………………………………………………………………………………………………………………………………………………………………………………………………………………………………………………………………………….

1. How important do you believe wastewater treatment works are for your community?
   - Very Important
   - Somewhat Important
   - Not Important
   - Unsure

**Section 3: Perceptions of Effectiveness**

1. To what extent do you believe the wastewater treatment facility in your community effectively treats wastewater?
   - Very effective
   - Somewhat effective
   - Not effective at all
   - Unsure
2. Have you ever noticed any negative impacts (e.g., odours, pollution) that you believe might be related to the wastewater treatment facility?
   - Yes
   - No
   - Unsure (Please elaborate if you answered yes)
3. How confident are you in the safety of the water discharged from the wastewater treatment facility?
   - Very confident
   - Somewhat confident
   - Not confident at all
   - Unsure
4. How important is it to you that wastewater treatment facilities remove plastic effectively?

- Very important
- Somewhat important
- Neutral
- Somewhat unimportant
- Not important at all

1. Do you know about the presence of plastic pollutants in wastewater?

- Yes
- No
- Not sure

1. How do you think plastic pollutants enter wastewater?

- Industrial discharge
- Domestic waste
- Stormwater runoff
- Other (please specify):…………………………………………………………..

1. In your opinion, how effective do you think current wastewater treatment plants are in removing plastic pollutants?

- Very effective
- Moderately effective
- Ineffective
- Not sure

1. What factors do you think influence the efficiency of wastewater treatment plants in removing plastic pollutants? (**Check all that apply**)

- Technology used
- Funding and resources
- Government regulations
- Public awareness and engagement
- Other (please specify):…………………………………………………………..

1. What factors do you believe contribute to the efficiency or inefficiency of wastewater treatment in removing plastic pollutants?

…………………………………………………………………………………………………………………………………………………………………………………………………………………………………………………………………………………………………………………………………………………………………………

1. How concerned are you about the presence of plastic pollutants in wastewater?

- Very concerned
- Somewhat concerned
- Not concerned
- Unsure

1. What do you think should be the top priority for wastewater treatment facilities regarding plastic pollution removal?

…………………………………………………………………………………………………………………………………………………………………………………………………………………………………………………………………………………………………………………………………………………………………………

1. Do you think the public has a role to play in reducing plastic pollution in wastewater?

- Yes, individuals can make a difference
- No, it's solely the responsibility of industries and governments
- Not sure

1. What measures do you think individuals can take to reduce plastic pollution in wastewater?

…………………………………………………………………………………………………………………………………………………………………………………………………………………………………………………………………………………………………………………………………………………………………………

1. Have you ever received information or education regarding plastic pollution in wastewater?

- Yes
- No

1. How effective do you think public awareness campaigns are in addressing plastic pollution in wastewater?

………………………………………………………………………………………………………………………………………………………………………………………………………………………………………………………………………………………………………………………………………………………………………….

1. How much trust do you have in the information provided by wastewater treatment authorities regarding plastic removal?

- Complete trust
- Moderate trust
- Limited trust
- No trust
